# P3Ka: A Driver of Tumor Metastasis?

**DOI:** 10.18632/oncotarget.162

**Published:** 2010-09-21

**Authors:** Leigh Zawel

**Affiliations:** Sanofi-Aventis Oncology Business Unit, Cambridge, Massachusetts, USA

The link between PI3K and cancer is among the strongest in the genome. PI3K sits in a critical node in cancer signaling space where it integrates growth and survival signals from RTKs and Ras to the mTOR, MAPK, FOXO1 and GSK3β signaling pathways. The Class I PI3Ks are dimeric kinases comprised of one regulatory subunit (p85 α, β, or γ) and one catalytic subunit (p110 α, β, or γ) which phosphorylate phosphoinositides at the D-3 position of the inositol ring in response to environmental cues. Constitutive activation of the PI3Ks is transforming in experimental models and in human breast, colon and endometrial cancer, activating mutations in the gene for PIK3CA (p110α) are seen in 30% of the patient population. Inactivating mutations to the p85 regulatory subunit have been described in a subpopulation (~10%) of colorectal cancer patients. Moreover, the PTEN phosphoinositide phosphatase, nature's brake to proliferative PI3K signaling, is a frequently mutated tumor suppressor gene [1, 2 and references therein].

Given the successes over the last decade in developing small molecule inhibitors targeting the BCR-ABL, EGFR, and BRAF oncogenic kinases, it is not surprising that numerous PI3K inhibitors have been developed and are currently being evaluated in human clinical trials. Despite the fact that among the p110 isoforms, p110α is preferentially mutated in cancer, the first wave of PI3K inhibitors to enter the clinic consisted of non-isoform selective (pan) molecules. While these inhibitors are broadly active in preclinical cancer models [3, 4] concern as to whether the lack of selectivity may adversely impact the therapeutic index has led to follow up efforts to develop isoform-selective PI3K inhibitors.

Working from an imidazopyridine-based scaffold, Schmidt-Kittler et al optimized PI3Kα-selective inhibitors with nM potency against PI3Kα (J-series). Among class I PI3Ks (α, β, γ, δ) the J-series compounds averaged 20 to 30 fold selectivity in favor of the p110α isoform in vitro but did not discriminate between wt and mutant (E545K, H1047R) forms of the enzyme. Representative compounds from the series inhibited growth and phospho-AKT levels in HCT116 cells in the 100 nM range in vitro, independent of PI3K mutational status. An unexpected finding occurred during the in vivo evaluation of two such compounds from the series. Although phospho-AKT levels were robustly inhibited in HCT116 xenograft tumor tissue, this translated into only modest effects on tumor growth inhibition. In contrast, when the compounds were evaluated in a metastatic version of the HCT116 model, wherein tumor cells injected into the spleen spread to the liver and lungs, the emergence of distant metastases was significantly curtailed [5]. The conclusions from this work — that PI3Kα inhibitors may be more useful for limiting the spread of a primary tumor to distant sites than treating established tumors — would be strengthened by additional studies incorporating isoform specific shRNAs in one or more in vivo tumor models. Still, the findings are provocative and raise many interesting questions.

## Why does in vitro anti-proliferative activity not translate to the in vivo setting?

J124 and J128 displayed potent inhibition of HCT116 growth in vitro in the nM range. Arcaro and coworkers also reported single agent activity in vitro with PI3Kα-selective inhibitors in a panel of medulloblastoma cell lines [6].

Assuming intra-tumoral levels of the J-series compounds reached exposures equivalent to those assessed in vitro (~100nM), why was potent anti-proliferative activity against the primary tumor not observed despite effective modulation of the phospho-AKT pharmacodynamic marker? The data suggest that modulation of phospho-AKT is insufficient for in vivo anti-tumor activity and beg the question — what PI3Kα-dependent processes impact the spread of the primary tumor? One also can't help but wonder whether p110α mutational status impacts the metastatic potential of a tumor.

## What is the mechanism of action through which PI3Kα regulates metastasis?

PI3Ks can phosphorylate both lipid and protein substrates. It is tempting to speculate that the modulation of as yet undefined substrates regulating angiogenesis, cell adhesion or migration accounts for these observations. Identification of these biomarkers will be essential for successful clinical evaluation of these compounds.

## How should α-selective inhibitors be evaluated in the clinic?

If indeed the utility of such compounds lies in preventing cancer spread, the best setting for single agent treatment may be in situations where measurable disease is not an issue (i.e. surgically removed or irradiated) and prevention of new lesions is needed. While first or second line treatment of advanced or metastatic disease may benefit patients by virtue of preventing new lesions, the ideal treatment setting is likely as an adjuvant therapy in patients with initially resectable localized disease with high metastatic recurrence rates. A potential endpoint would be progression free survival following surgery in patients receiving PI3Kα inhibitor therapy vs. standard of care adjuvant chemotherapy.

PI3Kα inhibitor therapy may also be well suited for use in combination with other targeted or cytotoxic agents, as has been shown for pan PI3K inhibitors and MEK inhibitors in models of basal-like breast cancer [7].

In closing, the findings of Schmidt-Kittler et al leave fertile ground for exploratory research in numerous directions. The emergence of PI3Kα-selective tool compounds should catalyze future discoveries that can inform the medical community on the optimal use of such agents upon their arrival in the clinic.
